# pH-Dependent Conformational Changes in the HCV NS3 Protein Modulate Its ATPase and Helicase Activities

**DOI:** 10.1371/journal.pone.0115941

**Published:** 2014-12-31

**Authors:** Gustavo Tavares Ventura, Emmerson Corrêa Brasil da Costa, Anne Miranda Capaccia, Ronaldo Mohana-Borges

**Affiliations:** Laboratório de Genômica Estrutural, Instituto de Biofísica Carlos Chagas Filho, Universidade Federal do Rio de Janeiro, Rio de Janeiro, RJ, Brazil; Wuhan University, China

## Abstract

The hepatitis C virus (HCV) infects 170 to 200 million people worldwide and is, therefore, a major health problem. The lack of efficient treatments that specifically target the viral proteins or RNA and its high chronicity rate make hepatitis C the cause of many deaths and hepatic transplants annually. The NS3 protein is considered an important target for the development of anti-HCV drugs because it is composed of two domains (a serine protease in the N-terminal portion and an RNA helicase/NTPase in the C-terminal portion), which are essential for viral replication and proliferation. We expressed and purified both the NS3 helicase domain (NS3hel) and the full-length NS3 protein (NS3FL) and characterized pH-dependent structural changes associated with the increase in their ATPase and helicase activities at acidic pH. Using intrinsic fluorescence experiments, we have observed that NS3hel was less stable at pH 6.4 than at pH 7.2. Moreover, binding curves using an extrinsic fluorescent probe (bis-ANS) and ATPase assays performed under different pH conditions demonstrated that the hydrophobic clefts of NS3 are significantly more exposed to the aqueous medium at acidic pH. Using fluorescence spectroscopy and anisotropy assays, we have also observed more protein interaction with DNA upon pH acidification, which suggests that the hydrophobic clefts exposure on NS3 might be related to a loss of stability that could lead it to adopt a more open conformation. This conformational change at acidic pH would stimulate both its ATPase and helicase activities, as well as its ability to bind DNA. Taken together, our results indicate that the NS3 protein adopts a more open conformation due to acidification from pH 7.2 to 6.4, resulting in a more active form at a pH that is found near Golgi-derived membranes. This increased activity could better allow NS3 to carry out its functions during HCV replication.

## Introduction

The hepatitis C virus (HCV) is a major cause of chronic liver disease with an estimated 170 million people infected worldwide. HCV can be classified into 7 major genotypes (1-7), differing in their nucleotide sequence by 30–35% [1]. Genotype 1 is the most prevalent worldwide and is also the least responsive to the standard of care (SOC) treatment. Patients with persistent infections are at high risk to develop serious liver damage, including steatosis, fibrosis, cirrhosis and even hepatocellular carcinoma [2]. No vaccine against HCV is available, and the usual SOC treatment consists of weekly injections of pegylated interferon (PEG-IFN) plus daily oral doses of ribavirin (RBV) [3]. In HCV genotype 1 infected patients, promising results were reported with the addition of the direct acting antivirals (DAAs) boceprevir [4] and telaprevir [5]. These DAAs were added to the SOC treatment, thereby increasing sustained virological response (SVR) rates from less than 50% to 70% in patients treated with a combination of PEG-IFN, RBV and one of the DAAs [6]. However, due to significant side effects and high costs, many patients abandon the therapy [7], [8]. Recently, three new HCV DAAs were approved in the United States and Europe: simeprevir, sofosbuvir and ledipasvir. With the addition of simeprevir and sofosbuvir to the treatment, SVR rates of the order of 90% or more could be achieved even against genotype 1 infected patients [9]. The same SVR rates were achieved with the addition of ledipasvir, which also started to be used in combination with the other DAAs for the emergence of all-oral IFN-free HCV treatments. This combination makes possible to achieve increased SVR rates with much less side effects for the patients, being considered the most viable option for the eradication of HCV nowadays [10]. However, even considering all these new options for the treatment, a constant search for and development of highly specific and efficient drugs to combat HCV infection is still necessary, because patients have to deal with side effects and the high costs of the treatments using these new DAAs.

HCV belongs to the Hepacivirus genus within the Flaviviridae family, which also includes West Nile, yellow fever and dengue virus [11]. The HCV genome consists of a positive-sense single strand uncapped RNA of ∼9.6 kb, which is translated into a ∼3000-amino acid polyprotein precursor at the host cell's rough endoplasmic reticulum [12]. After being processed by cellular and viral proteases, the large polyprotein is cleaved into three structural proteins (core, envelope proteins E1 and E2) that will form the viral particles, the small hydrophobic p7 protein and six nonstructural (NS) proteins (NS2, NS3, NS4A, NS4B, NS5A and NS5B). These nonstructural proteins are involved with the viral life cycle and replication [13]. Among the NS proteins, NS3 is considered one of the most important for drug development because it is a multifunctional protein composed of a serine protease N-terminal domain and an RNA helicase/NTPase C-terminal domain [14]. Both enzymatic activities have been well characterized and high resolution structures of the protein have been solved [15]. The protease domain of NS3 has emerged as a prime target for the development of DAAs [16] and, although it is appealing as a good antiviral target, specific inhibitors against the NS3 helicase domain are poorly described and numerically insignificant compared to those against the protease domain [14], [17].

Studies have demonstrated that the NS3 helicase is essential for viral replication, both in whole animal [18] and replicon models [19], [20], validating this domain as a suitable target for drug development. HCV NS3 helicase's most probable major function is to assist in replication of the viral RNA by tracking along RNA and resolving double-stranded RNA intermediates formed during this process [17]. This proposed function has become more evident with the discovery of NS3's capacity to stimulate NS5B to synthesize long RNAs [21]. One interesting peculiarity of HCV NS3 helicase is that its activity is optimized at a relatively acidic pH of 6.5 [22]–[25]. As replication of the HCV RNA occurs, the replication complex moves from the endoplasmic reticulum (ER) to the Golgi apparatus, resulting in local cellular pH changes. The Golgi pH is typically ∼6.4, in contrast to the pH∼7.2 of the ER [26], meaning that pH acidification could somehow structurally modify NS3 so that it can perform a possible role in virus maturation or particle assembly [27], [28]. In addition, it has been previously shown that the membranous webs where HCV RNA replication occurs may contain components of the Golgi apparatus [12], [29], further suggesting the importance of pH acidification in NS3's helicase role during RNA synthesis by the RNA-dependent RNA polymerase NS5B. Although it is known that important structural changes that modulate enzymatic activity occur in the dengue virus NS3 helicase [30], they have not yet been described for HCV NS3, especially in the context of acidification during RNA replication. In this context, the aim of this work was to investigate structural changes of the NS3 protein as a function of pH using biophysical techniques and to correlate these changes with its enhanced ATPase and helicase activities at acidic pH. We observed that the helicase domain adopts a less stable structure at pH 6.4 and that the hydrophobic effect could be an important factor for the increase in ATPase activity and DNA binding at this pH. Our results suggest that the NS3 protein adopts a more open conformation due to acidification from 7.2 to 6.4 and is more active at a pH similar to that found near Golgi-derived membranes. These conformational changes could allow NS3 to better carry out its functions during HCV replication.

## Results

It is largely known that pH is an important physicochemical property for the regulation of enzymatic activities. In the particular case of HCV, the multifunctional NS3 protein has optimal ATPase and helicase activities at approximately pH 6.5, while a significant reduction in these enzymatic activities is observed at approximately pH 7.2 [22]–[25]. In this work, the truncated NS3 helicase domain (NS3hel) and the full-length NS3 protein (NS3FL) from the HCV 1b genotype were purified from *E. coli* extracts to investigate the changes in NS3 structure under acidic conditions.

### Effect of pH acidification on the NS3 intrinsic fluorescence

**Figure 1:**
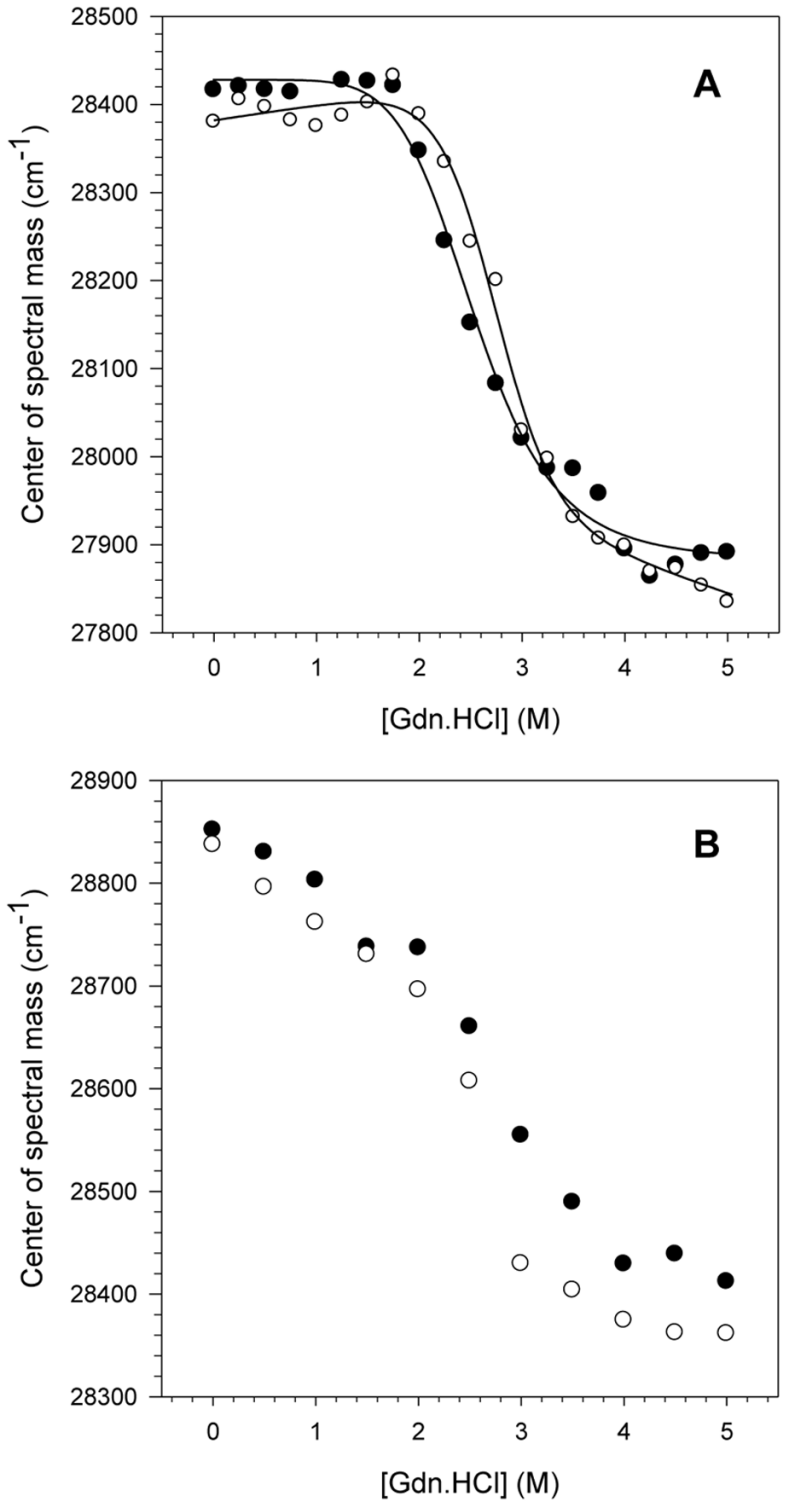
pH effects on the NS3 tertiary structure upon chemical denaturation. The CM values obtained for NS3hel (A) and NS3FL (B) were calculated at pH 6.4 (closed circles) and 7.2 (open circles) upon increasing Gdn.HCl concentrations using Equation 1 (Material and Methods). The fluorescence spectra were obtained at 25°C and assay buffers were composed of 50 mM MOPS-NaOH (pH 6.4 or 7.2), 200 mM NaCl, 5 mM β-mercaptoethanol and 5% glycerol. The protein concentration was 1 µM.

The structural stability of NS3hel and NS3FL was evaluated at pH 6.4 and 7.2 using chemical denaturation by increasing guanidine hydrochloride (Gdn.HCl) concentrations. The intrinsic fluorescence of the 6 tryptophan (Trp) residues present in the NS3 sequence (4 and 2 in the helicase and protease domains, respectively) was monitored (Fig. 1). It was observed that both constructs denatured and lost their tertiary structures upon increasing the Gdn.HCl concentration from 0 to 5 M. This loss of tertiary structure was monitored by determining the center of spectral mass (CM) for both constructs, which decreased as the protein denatured. A decrease in the CM indicates a significant red-shift effect on the emission of the Trp residues, which was, in this case, caused by solvent exposure during the denaturation process. The CM difference between the native (0 M Gdn.HCl) and denatured (5 M Gdn.HCl) conformational states measured for the NS3hel protein at both pH 6.4 and 7.2 were 28,400 and 27,850 cm^−1^ (ΔCM = 550 cm^−1^) (Fig. 1A), respectively, whereas the CM decreased from 28,850 to 28,350 cm^−1^ (ΔCM = 500 cm^−1^) for the NS3FL protein at the same pH values and denaturant concentrations (Fig. 1B). The Gibbs free energy variation (ΔG) was calculated for NS3hel only because the shape curves of the NS3FL denaturation could not be fitted properly. Moreover, the NS3hel denaturation process was reversible for this construct, which allows for accurate calculation of ΔG. Our results showed that NS3hel is more stable at pH 7.2 (ΔG_H2O_ = 5.6 kcal.mol^−1^, m = 2.07 kcal.mol^−1^M^−1^) than at pH 6.4 (ΔG_H2O_ = 3.21 kcal.mol^−1^, m = 1.44 kcal.mol^−1^M^−1^). Moreover, the parameter G_1/2_ that corresponds the Gdn.HCl concentration necessary to denature 50% of protein population was obtained for both proteins, and we observed that NS3hel presented a lower G_1/2_ value at pH 6.4 than at pH 7.2 (G_1/2_ = 2.5 M and 2.8 M, respectively), whereas NS3FL did not present any significant change (G_1/2_ = 2.5 M at both pHs). Another important fact is that the constructs clearly had different denaturation curve patterns. NS3hel exhibited a cooperativity plateau at the beginning of the denaturation process (from 0 to 2 M Gdn.HCl), whereas NS3FL showed a constant CM decrease until complete denaturation, which occurred at approximately 5 M Gdn.HCl. These differences can most likely be attributed to the exposure of the two Trp residues located in the protease domain, indicating that this domain is most likely less stable and starts denaturing before the helicase domain. In addition, NS3FL seems to form molten globule states at the beginning of the denaturation process, as is observed in its denaturation curve monitored by the extrinsic probe bis-ANS (data not shown). Nonetheless, at Gdn.HCl concentrations higher than 2 M both domains could equally contribute to the denaturation process, resulting in a pronounced decrease in the CM values observed between 2 and 4 M Gdn.HCl until the denaturation of their tertiary structures is complete.

### Effect of the acidic pH on the Trp residues exposure of the NS3 protein

**Figure 2:**
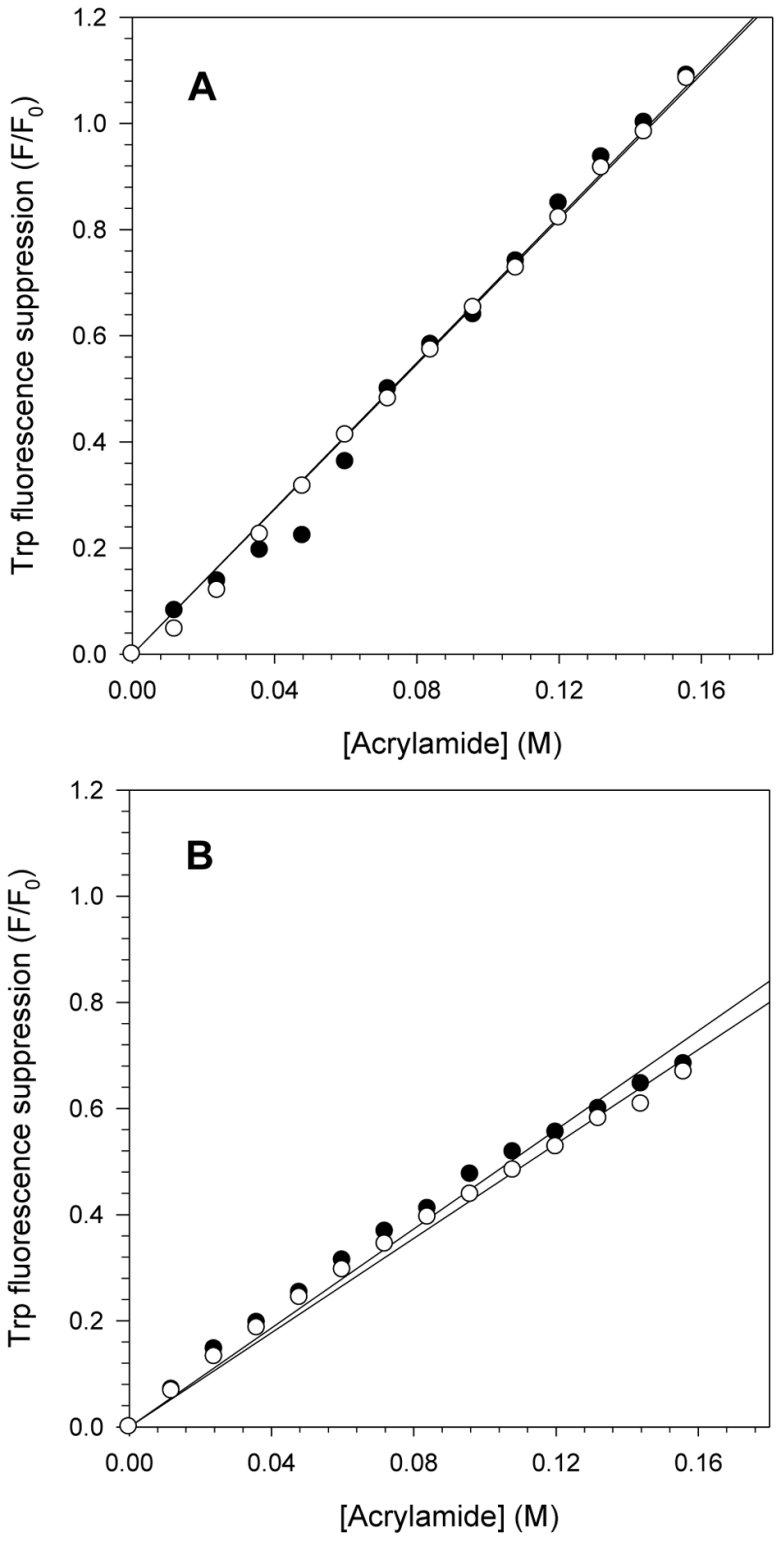
Fluorescence quenching of NS3 Trp residues by acrylamide at pH 6.4 and 7.2. Acrylamide concentrations ranging from 0 to 156 mM were used to monitor the exposure of the Trp residues of NS3hel (A) and NS3FL (B) at pH 6.4 (closed circles) and 7.2 (open circles) and to calculate the Stern-Volmer constant (K_sv_) using Equation 3 (Material and Methods). Each point corresponds to the mean of tryptophan fluorescence quenching by acrylamide obtained in three independent experiments. Spectra were acquired at 25°C in buffer solutions composed of 50 mM MOPS-NaOH (pH 6.4 or 7.2), 200 mM NaCl, 5 mM β-mercaptoethanol and 5% glycerol. The protein concentration was 1 µM.

The pH effect on the NS3hel and NS3FL tertiary structures was also assessed by suppressing the intrinsic Trp fluorescence using acrylamide as an external quencher. This experiment provides important information on the exposure of Trp residues at different conditions, such as pH. The Trp fluorescence of NS3hel and NS3FL at pH 6.4 and 7.2 was monitored by increasing the acrylamide concentrations to obtain the Stern Volmer constants (K_sv_) to measure the exposure of the Trp residues (Fig. 2). As expected, the Trp fluorescence quenching for both proteins increased linearly with acrylamide concentration. However, no significant differences were observed between the K_sv_ values at pH 6.4 and 7.2 for either protein (K_sv_ = 6.87 M^−1^ at pH 6.4 and 6.83 M^−1^ at pH 7.2 for NS3hel and K_sv_ = 4.67 M^−1^ at pH 6.4 and 4.44 M^−1^ at pH 7.2 for NS3FL). One important difference was the fluorescence quenching was more pronounced for NS3hel than for NS3FL, suggesting that the protease domain most likely adopts a more closed/compact conformation and/or promotes significant conformational changes in the helicase domain, thus preventing acrylamide binding, at this pH range. In other words, the quenching effect observed in NS3FL might be associated with the Trp located in the protease domain, or the presence of the protease domain may block acrylamide binding to the Trp located in the helicase domain.

### Effect of acidification on NS3 secondary structure

As we observed that the tertiary structure of NS3hel was less stable at pH 6.4 than at pH 7.2, it was plausible that the secondary structure of NS3 might also be altered. Secondary structure can be monitored during chemical denaturation using circular dichroism (CD) spectroscopy. This experiment was not performed with NS3FL because it could not be obtained at the high concentration necessary for the experiments due to aggregation problems.

**Figure 3:**
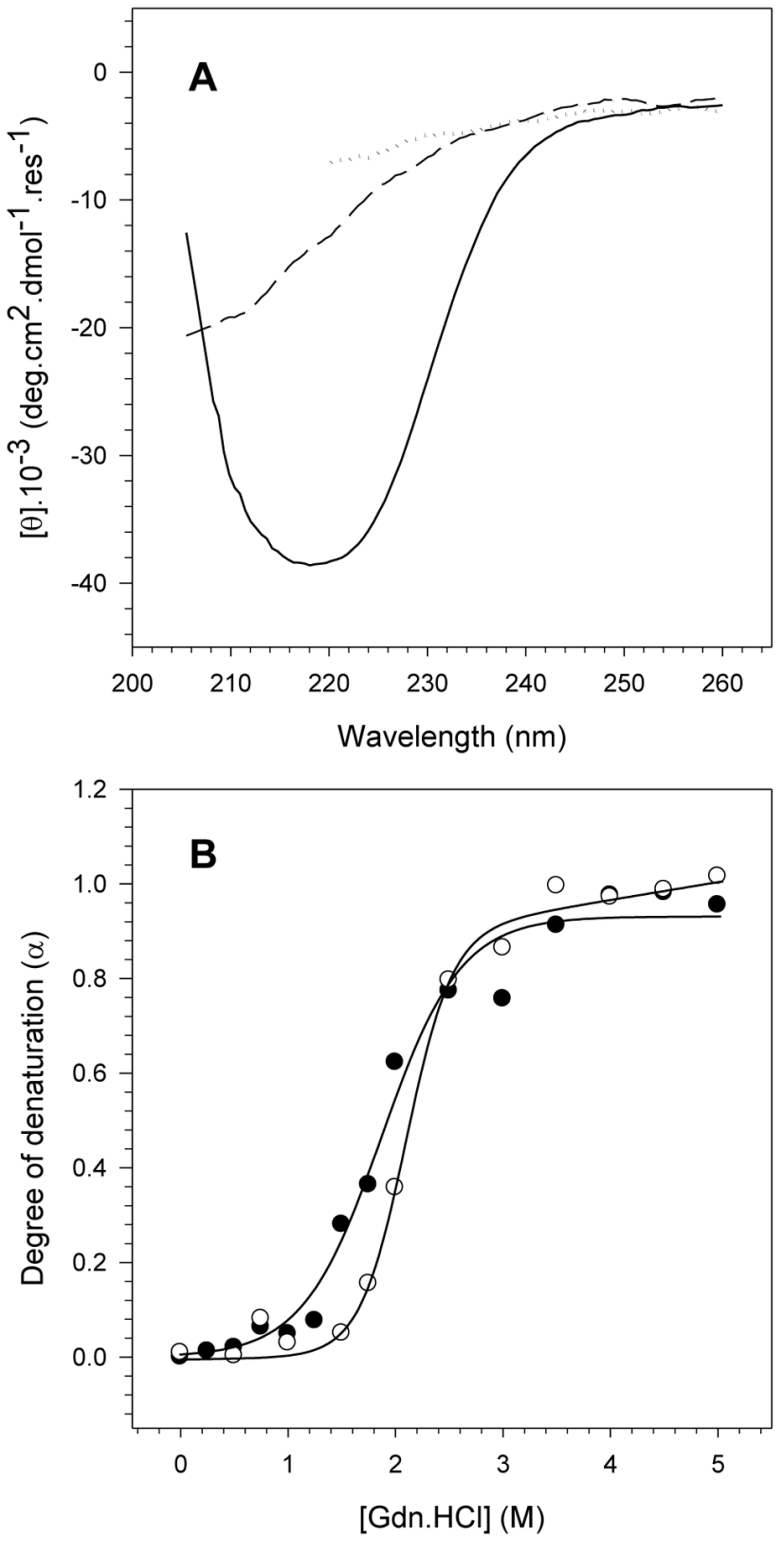
Analysis of the pH effects on the NS3 secondary structure upon chemical denaturation. A) CD spectra of 10 µM NS3hel acquired at pH 7.2 in the absence of Gdn.HCl (solid line), or presence of 2.5 M (dashed line) and 5 M (dotted line) Gdn.HCl. The spectra were the average of three scans after subtracting the buffer baselines. Each spectrum was converted into molar ellipticity using Equation 4 (Material and Methods). B) The ellipticity values at 222 nm (θ_222_) at each Gdn.HCl concentration (from 0 to 5 M) were used to compare the secondary structure stability of NS3hel at pH 6.4 and 7.2 and to calculate the degree of denaturation using Equation 5 (Material and Methods). Closed (pH 6.4) and open circles (pH 7.2) represent the degree of denaturation at each Gdn.HCl concentration. Spectra were acquired at 25°C in buffer solutions composed of 50 mM MOPS-NaOH (pH 6.4 or 7.2), 200 mM NaCl, 5 mM β-mercaptoethanol and 5% glycerol. The protein concentration was 10 µM.

As shown in Fig. 3A, NS3hel presented a typical α-helix + β-sheet CD spectrum at native conditions (in the absence of Gdn.HCl), which is in agreement with the three-dimensional structure already solved at high resolution for NS3hel [31], [32]. The CD spectra of NS3hel were also acquired as a function of the increasing Gdn.HCl concentration, and the ellipticity signal at 222 nm was converted into degree of denaturation (α) according to Equation 4 (Fig. 3B). A typical sigmoidal denaturation curve corresponding to the loss of secondary structure of NS3hel upon increasing Gdn.HCl concentration was observed for experiments performed at both pH 6.4 and 7.2. Moreover, NS3hel was less stable at pH 6.4 than at pH 7.2, as observed by ΔG (ΔG_H2O_ = 4.8 kcal.mol^−1^ and 5.8 kcal.mol^−1^, respectively) and G_1/2_ parameters (G_1/2_ = 1.82 M and 2.22 M, respectively). This loss of secondary structure stability at acidic pH is in accordance with our results obtained for the tertiary structure and could be related to the ATPase and helicase activity enhancement observed for NS3hel at this pH.

### Effect of the acidic pH on the hydrophobic clefts of the HCV NS3 protein

**Figure 4:**
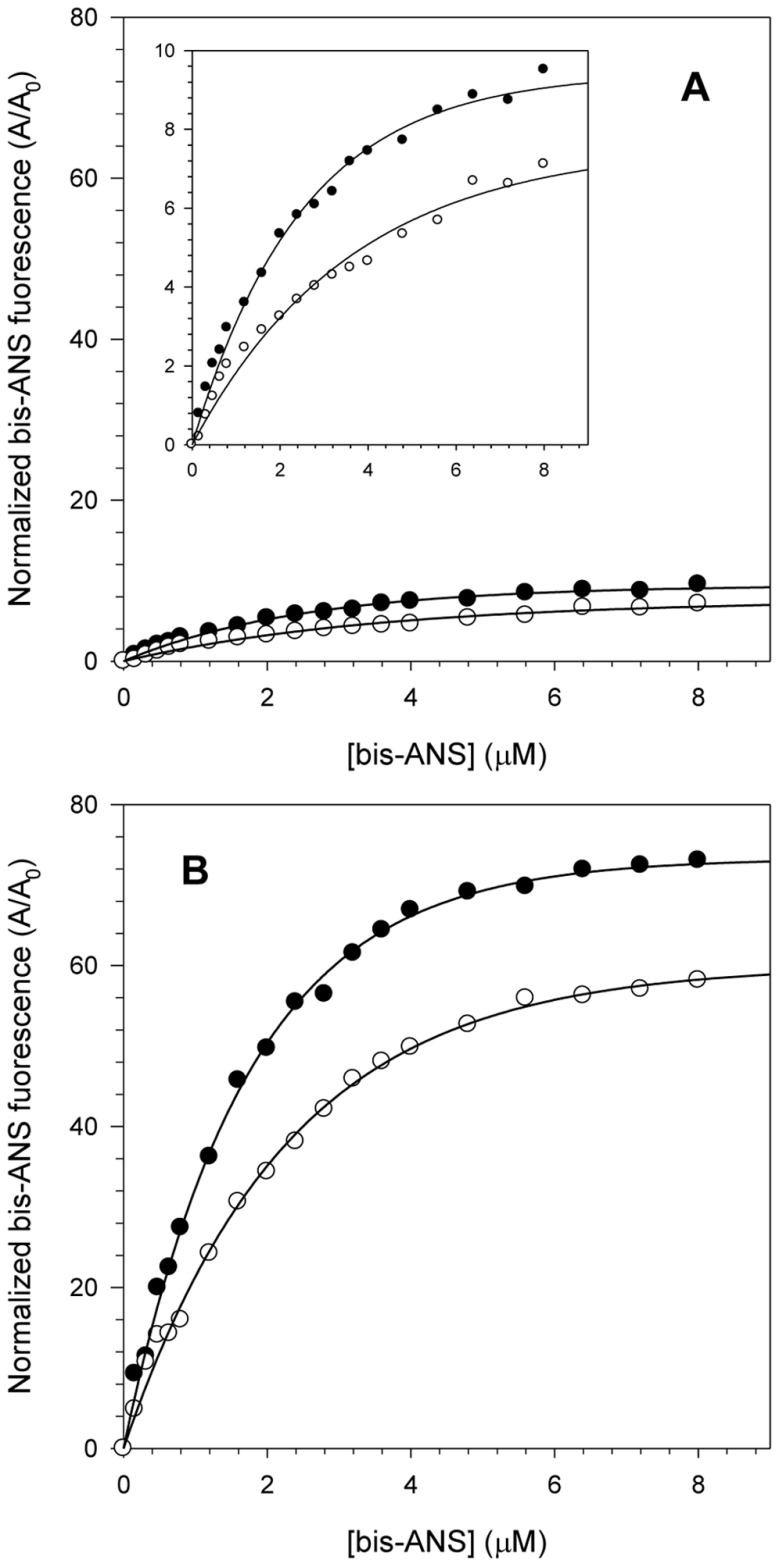
Interaction of the fluorescent extrinsic probe bis-ANS with NS3 at pH 6.4 and 7.2. bis-ANS concentrations ranging from 0 to 8 µM were used to compare NS3hel (A) and NS3FL (B) hydrophobic clefts exposure at pH 6.4 (closed circles) and 7.2 (open circles). The inset in the graph A shows a reduction in the y-axis scale to demonstrate more clearly the effect of increasing bis-ANS fluorescence at both pH. Each point corresponds to the mean of the normalized bis-ANS fluorescence intensity obtained in three independent experiments. Spectra were acquired at 25°C in buffer solutions composed of 50 mM MOPS-NaOH (pH 6.4 or 7.2), 200 mM NaCl, 5 mM β-mercaptoethanol and 5% glycerol. The protein concentration was 1 µM.

The extrinsic probe bis-ANS (4,4′-dianilino-1,1′-binaphthyl-5,5′-sulfonate) has been extensively used to provide important information about the exposure of hydrophobic clefts of proteins in different solution conditions because its fluorescence emission increases considerably upon binding to such clefts. To further understand the effect of acidic pH on the structure of NS3hel and NS3FL proteins, the fluorescence spectra of bis-ANS at both pH 6.4 and 7.2 upon binding those proteins were monitored, and these bis-ANS binding curves were used to obtain association constants (K_a_) at the different pHs (Fig. 4). Both NS3hel and NS3FL presented a pronounced increase in the exposure of their hydrophobic clefts at pH 6.4 as determined by an increase in the area under the bis-ANS emission spectra relative to its spectrum in buffer solution. This effect was much more pronounced for NS3FL (K_a_ = 0.58 µM^−1^ and 0.4 µM^−1^ at pH 6.4 and 7.2, respectively) than for NS3hel (K_a_ = 0.36 µM^−1^ and 0.22 µM^−1^ at pH 6.4 and 7.2, respectively). This difference between NS3hel and NS3FL is very likely a consequence of the presence of the protease domain in the full-length protein, which would increase the number of bis-ANS binding sites. Moreover, these results suggest that acidification most likely makes NS3 adopt a more open conformation, which could facilitate substrate binding, enhance the capability of NS3 to hydrolyze ATP and increase NS3's helicase activity.

### Association of the hydrophobic effect with the ATPase activity at acidic pH

**Figure 5:**
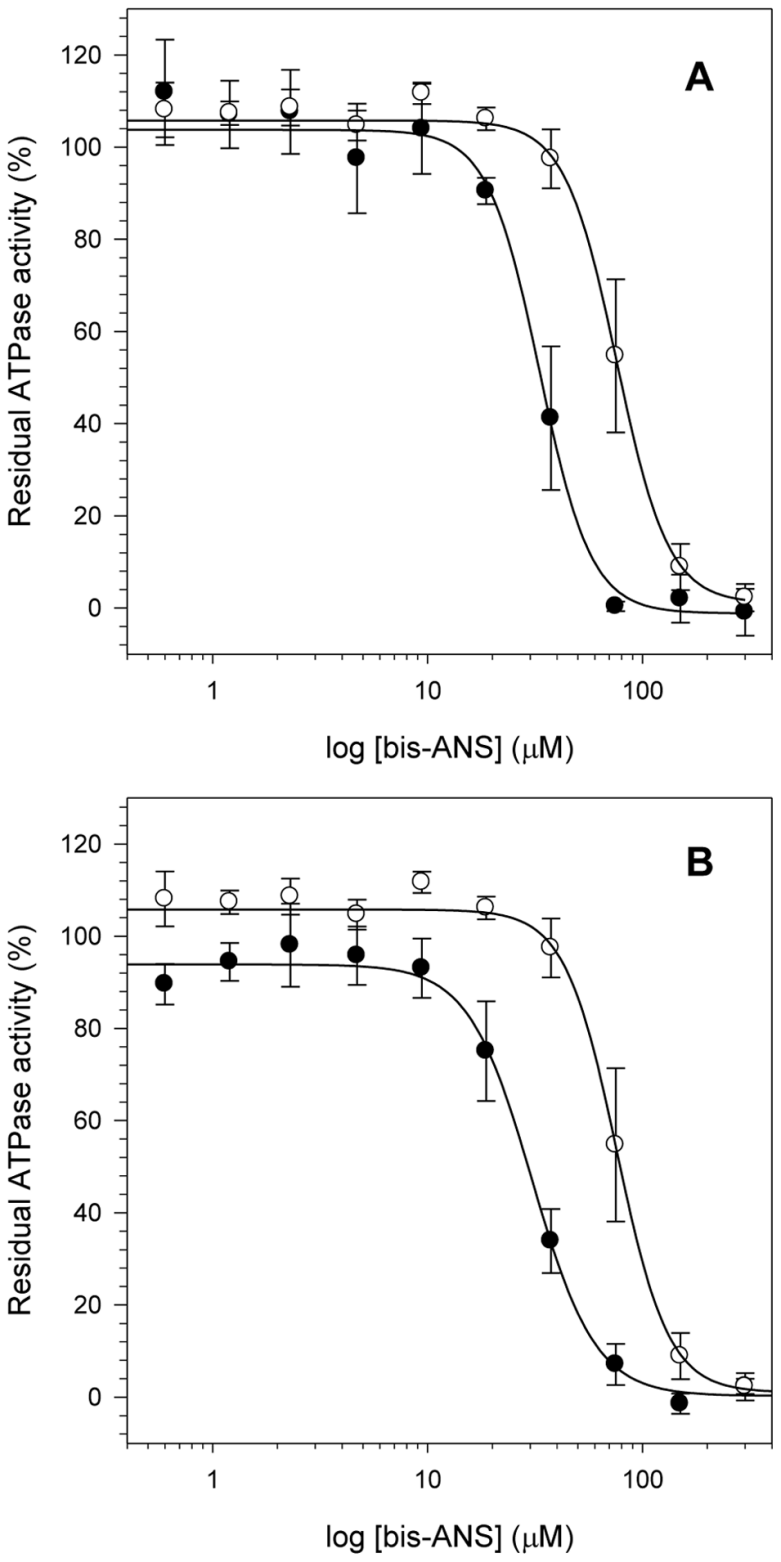
Effects of bis-ANS binding on ATPase activity at different pHs. Increasing bis-ANS concentrations (from 0 to 300 µM) were used to compare the effect of bis-ANS binding on the ATPase activity of NS3hel (A) and NS3FL (B). Closed (pH 6.4) and open circles (pH 7.2) represent the mean of the residual ATPase activity obtained in three independent experiments and bars indicate the standard error. Reactions were performed at 30°C during 60 min using 40 mM MES-KOH (pH 6.4) or Tris-HCl (pH 7.2), 5 mM DTT, 5 mM MgCl_2_, 100 mM KCl, 1 mM ATP and 0.1 µM of purified proteins. IC_50_ values were calculated using *Sigma plot* ver. 10.0 after plotting the dose-response curve of bis-ANS concentration versus residual ATPase activity.

The large increase of NS3 hydrophobic clefts exposure combined with the decrease in its stability upon acidification suggests that NS3 could adopt a more open conformation, which would likely affect its enzymatic activity. To confirm this hypothesis, we performed an assay to evaluate the effect of bis-ANS binding on the ATPase activity of NS3hel and NS3FL at pH 6.4 and 7.2 (Fig. 5). Binding of bis-ANS clearly reduced the ATPase activity both for NS3hel (Fig. 5A) and NS3FL (Fig. 5B) by competing for the ATP binding site. Inhibition by bis-ANS was much more pronounced at pH 6.4 (IC_50_ = 33±2 µM for NS3hel and 30±2 µM for NS3FL) than at pH 7.2 (IC_50_ = 76±4 µM for NS3hel and 75±4 µM for NS3FL). These results suggest that bis-ANS binding to the regions near the ATP binding site, which is located between subdomains 1 and 2 of NS3hel, occurs more at the acidic pH than at the higher pH. They also confirm the hypothesis that NS3 adopts a more open conformation and exposes more of its hydrophobic clefts at the lower pH, which would most likely favor ATP binding and, consequently, stimulate ATPase activity.

### Effect of pH acidification on DNA binding by NS3

**Figure 6:**
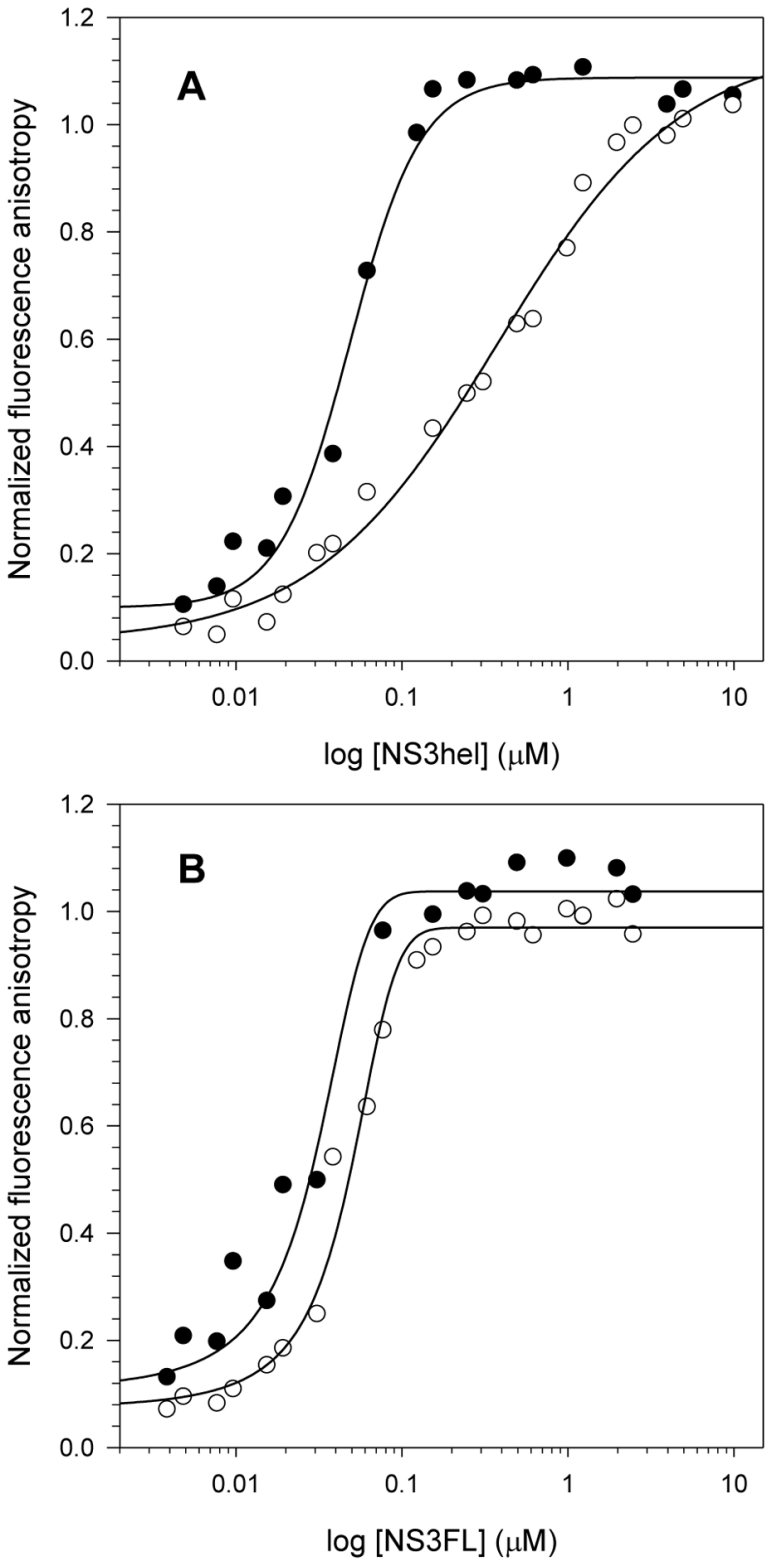
Effects of pH on NS3 binding to ssDNA. Increasing protein concentrations (from 0 to 10 µM NS3hel and 0 to 5 µM NS3FL) were used to compare NS3hel (A) and NS3FL (B) binding to a fluorescently-labeled ssDNA at pH 6.4 and 7.2 and to calculate the dissociation constants (K_d_) between ssDNA and the constructs. Closed (pH 6.4) and open circles (pH 7.2) represent the mean of fluorescence anisotropy obtained in three independent experiments. Data were obtained at 25°C and assay buffers contained 25 mM MOPS-NaOH (pH 6.4 or 7.2), 2 mM MgCl_2_ and 25 nM of the fluorescently labeled ssDNA.

As the pH clearly influences the ATPase activity of NS3 through important changes in hydrophobic cleft exposure, it was speculated whether DNA binding could also be affected by pH. For this purpose, a fixed concentration of a fluorescently labeled single-stranded DNA (ssDNA) was titrated with increasing concentrations of NS3hel and NS3FL at pH 6.4 and 7.2, and the fluorescence anisotropy of the ssDNA molecule was monitored (Fig. 6). The NS3hel protein-DNA interaction curves revealed differences. A higher anisotropy signal was observed at pH 6.4, even at low protein concentrations, and the highest signal was obtained near 250 nM protein. Conversely, at pH 7.2 the anisotropy reached these signals only at high protein concentrations (2 µM). NS3FL showed similar protein-DNA interaction curves, albeit with higher anisotropy signals at pH 6.4 at the low protein concentrations. NS3FL curves from both pHs reached the highest anisotropy signal near 150 nM protein. Interestingly, acidification seemed to be more important for NS3hel, as there was a drastic difference in the K_d_ obtained at both conditions (K_d_∼42 nM and 254 nM at pH 6.4 and 7.2, respectively). In contrast, a much smaller difference was observed for NS3FL (K_d_∼28 nM and 49 nM at pH 6.4 and 7.2, respectively). This effect is most likely related with the presence of other nucleic acid binding sites on NS3FL, as described elsewhere [14], [33]. The anisotropy values observed at low protein concentrations were high for both constructs, suggesting that at acidic pH, NS3 preferentially binds ssDNA at the nucleic acid binding site located in the helicase domain. At higher NS3FL concentrations, the anisotropy values at both high and low pH are similar. Thus, at high concentrations, NS3FL most likely binds to the ssDNA using its other sites, which may not be affected by acidification.

### Effect of DNA binding on NS3 structure monitored by Trp quenching and bis-ANS

**Figure 7:**
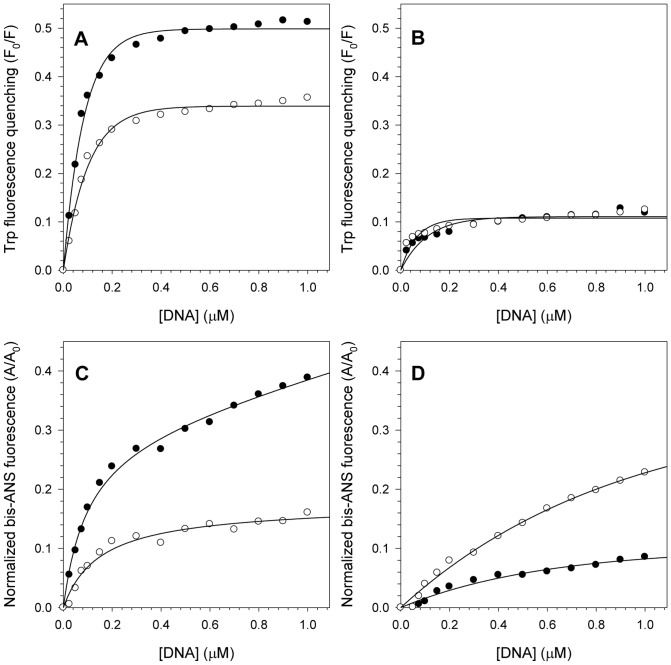
Effects of ssDNA binding on NS3 structure monitored by Trp fluorescence quenching and bis-ANS binding. Increasing ssDNA concentrations (from 0 to 1 µM) were used to compare the Trp fluorescence quenching of NS3hel and NS3FL (A and B, respectively) and the bis-ANS binding to these proteins (C and D, respectively) at pH 6.4 (closed circles) and 7.2 (open circles). Each point represents the mean of Trp fluorescence quenching or bis-ANS binding obtained in three independent experiments. Spectra were obtained at 25°C and assay buffers contained 25 mM MOPS-NaOH (pH 6.4 or 7.2), 2 mM MgCl_2_ and 1 µM of purified proteins.

To evaluate the structural changes caused by ssDNA interaction with NS3, Trp fluorescence quenching and bis-ANS binding experiments were carried out for both NS3 constructs at pH 6.4 and 7.2 with increasing ssDNA concentrations (Fig. 7). In these experiments, the protein concentration was fixed and the ssDNA concentration varied from 0 to 1 ΔM to evaluate the structural changes caused by protein-DNA interaction.

Based on the Trp fluorescence quenching upon ssDNA binding, NS3hel fluorescence suppression significantly increased at pH 6.4 relative to pH 7.2 (Fig. 7A), but no significant changes were detected for NS3FL between both pH values (Fig. 7B). Therefore, less fluorescence quenching was observed in NS3FL than in NS3hel, which clearly indicates that ssDNA is binding to another site on NS3FL other than the nucleic acid binding site located at NS3hel, because there is a key Trp residue located in this site [34] that should be quenched as a consequence of DNA binding.

When the bis-ANS fluorescence was analyzed, hydrophobic clefts exposure of both constructs increased with increasing ssDNA concentration. The hydrophobic clefts exposure of NS3hel clearly increased more upon ssDNA binding at pH 6.4 (Fig. 7C), whereas NS3FL showed an opposite effect with more hydrophobic clefts exposure occurring at pH 7.2 (Fig. 7D). This effect might also be associated with structural changes in the protease domain or the other nucleic acid binding sites in NS3FL, as was observed for the intrinsic fluorescence quenching results.

## Discussion

NS3 is one of the key proteins involved in HCV replication. Previous studies have demonstrated its importance in both whole animal [18] and replicon models [19], [20]. The protease domain of NS3 (NS3 pro) has been one of the main targets of anti-HCV drug design, and some of these compounds are either already being used in advanced clinical trial phases or in combination with the SOC treatment for genotype 1-infected patients [9], [35]. On the other hand, the NS3hel domain has not gained as much attention for drug development because it resembles the helicases and similar motor proteins encoded in all human cells [14]. Nonetheless, there has recently been great interest in finding inhibitors against this domain after the discovery of compounds the inhibited a helicase encoded by the herpes simplex virus (HSV) that relieved disease symptoms [36], [37].

An important factor to consider for drug design against NS3 is the pH dependence of its enzymatic activities. While its ATPase and helicase activities are optimal at relatively acidic pH (∼6.5) [22]–[25], the protease's optimal pH is 8.0, and pH changes in either direction result in significant loss of protease activity [38], [39]. It is possible that, although both domains reside within the same protein, each is active at a different stage of the replication cycle depending on the local pH environment [25]. The activity of each domain is most likely modulated by structural changes in the domains or in the whole NS3 protein. In this context, the conformational changes of NS3hel and NS3FL at pH 6.4 (Golgi) and 7.2 (ER) were monitored to evaluate whether the ATPase and helicase activities could be favored at an acidic pH.

Acidification appears to be an important factor for the overall stability of the tertiary structure of NS3hel. The ΔG and G_1/2_ values indicate that this construct is less stable at acidic pH. This effect was also confirmed by the evaluation of the secondary structure stability of NS3hel, which indicated that this domain presents a less stable secondary structure at pH 6.4 based on its increased susceptibility to chemical denaturant at low pH. In addition, NS3hel and NS3FL have different denaturation curves regardless of pH. These differences might be associated with the presence of the protease domain on NS3FL protein, which is most likely less stable than the helicase and starts its denaturation first, as can be seen in the NS3FL denaturation curve between 0 and 2 M Gdn.HCl. These results are in agreement with previous works that have shown by calorimetry and spectroscopy techniques that NS3pro is an intrinsically disordered domain [40], [41]. In addition, all 4 Trp residues found on NS3hel are located in the subdomain (SD) 3, which, along with SD 1, has already been demonstrated to be rigid. SD 2, however, is considered to be more flexible. This information is supported by works that demonstrated that NS3 could be found either on an open or closed conformation, with SD 2 freely rotating relative to SD 1 and 3 [31], [32].

The acrylamide quenching experiments did not show any significant differences between the two pH values, suggesting that the Trp residues are equally exposed to solvent for both constructs at both pHs. The only difference observed was in the level of fluorescence suppression, which was more pronounced for NS3hel than for NS3FL. This effect may be related to the two Trp residues located in NS3 pro, whose quenching signal may overshadow any quenching effects in the helicase domain. As we have already discussed before, NS3pro is an unstable and intrinsically disordered domain, which could favor acrylamide binding and, consequently, decrease quenching of the four Trp residues located in NS3hel. This occurrence would reduce the level of fluorescence quenching observed in NS3FL.

Binding curves with bis-ANS have shown significant increases in the hydrophobic clefts exposure of both constructs at acidic pH. This result suggests that NS3 most likely adopts a more open conformation due to pH acidification, which could favor bis-ANS binding as a result of the increased hydrophobic clefts exposure.

Lam and colleagues [25] have proposed model mechanism for NS3hel translocation along RNA that would be activated by acidic pH. In this model, NS3hel exists in two basic conformations, and the transition between the two states is regulated by ATP binding. In the absence of ATP, the protein tightly binds to the nucleic acid. Conversely, in the presence of ATP, the protein would bind weakly and then be able to slide along the nucleic acid. They also revealed that at low pH, the helicase-ATP complex bound nucleic acids 50-fold more tightly than at higher pH. At this low pH, they suggested that an ionizable side-chain, or several of them, can rotate into the nucleic acid binding cleft upon ATP binding.

Our results clearly show that bis-ANS preferentially binds both NS3hel and NS3FL at acidic pH and competitively inhibits ATP binding, as observed by its drastically reduced ATPase activity. Thus, the hydrophobic effect is important for ATP binding, suggesting that the charged residues are not the only residues that are important for substrate binding and nucleic acids translocation, as suggested before [25], [42]. Hydrophobic clefts exposure might also be directly associated with ATP binding and, thus, to the enzymatic activity enhancement at acidic pH.

The effect of acidification on DNA binding was also evaluated. Although no known DNA stage in the replication cycle of HCV has been observed, NS3hel unwinds DNA better than RNA [43]–[45]. Additionally, DNA molecules have been already used in previous works to characterize the interaction between NS3 and nucleic acids [25], [46], and some NS3-DNA complexes have even had structures solved [20], [34]. Fluorescence anisotropy assays with a fluorescently labeled ssDNA demonstrated that the interaction of NS3hel and NS3FL with DNA is pH-dependent. Although it has already been reported that acidification would not be an important factor for ssDNA binding to NS3hel without ATP bound [25], our results suggest the contrary. In addition, our data indicate that ssDNA most likely binds more than one site on NS3FL. Previous works have proposed that the interface between the protease and helicase domains could be an additional site for RNA binding because it is positively charged and could accommodate nucleic acids [14], [33], [47], [48]. Additionally, a recent work has shown that RNA could bind directly the protease domain, and ssDNA was reported to inhibit protease activity NS3 pro significantly while NS3FL was less affected. These results suggest that ssDNA preferentially binds to the NS3hel nucleic acid binding site [49]. Our results also corroborate these findings, because ssDNA could bind to the binding site on the NS3hel domain of NS3FL at low protein concentrations, thus generating the same effect on the anisotropy signal as was observed for NS3hel (high protein-DNA interaction at pH 6.4). At higher protein concentrations, ssDNA could bind the other sites, such as the interface between the domains or the protease domain itself. These domains were less affected by pH, resulting in similar anisotropy signals at pH 6.4 and 7.2.

To further investigate these findings, we performed intrinsic and extrinsic fluorescence experiments using increasing DNA concentrations at pH 6.4 and 7.2. While NS3hel clearly exhibited a significant increase in Trp fluorescence quenching at pH 6.4, NS3FL showed no changes. This result supports the hypothesis that DNA is binding to another site on NS3FL, as was observed by fluorescence anisotropy. One of the 4 Trp residues of NS3hel is located at the nucleic acid binding site, and it has already been shown that it is a key residue for nucleic acid binding because it stacks between nucleic acid bases [34]. Thus, nucleic acid binding to NS3hel should suppress Trp fluorescence, while binding to other sites would lessen the significance of this effect on the overall Trp fluorescence quenching, as was observed for NS3FL in this experiment.

Evaluation of the hydrophobic effect showed that both constructs increased their hydrophobic clefts exposure upon DNA binding, thus providing additional evidence for the hypothesis that NS3 adopts a more open conformation to favor ATP and DNA binding and enhance its enzymatic activities. For NS3hel, this effect was more evident at pH 6.4, while for NS3FL it was more evident at pH 7.2. This result might be once more related to the different nucleic acid binding sites on NS3FL; thus these other sites may be affected differently by pH acidification when compared to the site in NS3hel.

Aydin and colleagues have recently proposed a model in which they related the protease and helicase functions of NS3/4A (NS3FL plus the protease cofactor) [50]. Using mutational analysis and molecular dynamics simulations, they suggested that the interface between the protease and helicase domains may be important for nucleic acid binding. Furthermore, they proposed that NS3 could adopt compact or extended (open) conformations with the NS3hel's unwinding reaction involving a transition between these two different states. They also proposed that upon ATP or DNA binding, NS3 could adopt this extended conformation, which would be the appropriate conformational state for translocation and nucleic acid unwinding. Our findings lead us to a similar model, but we also consider the effects of acidification and the consequent increase in the hydrophobic clefts exposure as important factors that influence this transition between the two states even before ATP or nucleic acids binding.

Taken together, our results suggest the importance of pH-dependent conformational changes that cause NS3 to adopt a less stable and more open conformation with evident increases in hydrophobic clefts exposure. These pH-dependent conformational changes are important for both ATP and DNA binding, as well as for enhancement of NS3's enzymatic activities. We also propose that this hydrophobic effect might be considered as an important factor for drug development using NS3hel as a model, because local cellular pH changes during the replication process could affect the efficacy of drug compounds. Moreover, our data confirm the importance of different nucleic acid binding sites on NS3FL, which should also be considered during drug development that targets the helicase activity, as has already been suggested by other groups.

## Materials and Methods

### Reagents

Rosetta [λDE3] *E. coli* strain, pET21a and pET21d plasmids were purchased from Novagen (WI, USA). Ampicillin and chloramphenicol were obtained from USB (OH, USA). Isopropyl β-D-1-thiogalactopyranoside (IPTG), lysozyme, DNAse, p-methyl-sulfonylfluoride (PMSF), imidazole, Gdn.HCl, bis-ANS and adenosine 5′-triphosphate (ATP) were purchased from Sigma (MO, USA). The unlabeled and 5′-carboxyfluorescein (5-FAM)-labeled ssDNA oligo(dT)_20_ were purchased from IDT (IA, USA).

### Expression and purification of the constructs

Con1/SG-Neo (I) was used as template for the generation of constructs NS3hel and NS3FL. Con1/SG-Neo (I) contains the cDNA of the HCV 1b subgenomic replicon, which codes for the S1179I mutant in the NS5a sequence. The NS3hel gene was cloned in frame using the multiple cloning site of the *E. coli* expression vector pET21d-hisTev (a pET21d plasmid, which was modified to codify a 6x histidine tag followed by a cleavage site recognized by the *Tobacco Etch virus* nTev protease at the protein N-terminus) to construct the plasmid pET21d-hisTev-NS3hel. For NS3FL cloning, a pET21a plasmid that carries a C-terminal 6xhistidine tag was used.

Protein expression was carried out after transformation of competent Rosetta [λDE3] *E. coli* strain with the recombinant plasmids by heat shock. Transformed cells were selected on Luria-Bertani (LB) agar plates with ampicillin (100 µg/mL) and chloramphenicol (34 µg/mL). Single colonies from plates of freshly transformed Rosetta [λDE3] cells were used to initiate growth of the constructs, which was carried out at 37°C in LB medium supplemented with ampicillin (100 µg/mL) and chloramphenicol (34 µg/mL). When the cell density reached an OD_600_ of 0.8–1.0, the temperature was lowered rapidly to 30°C and recombinant proteins expression was induced with 0.5 mM IPTG. Four hours after induction, cells were harvested and frozen at −80°C prior to purification.

All purification steps were performed at 4°C. Cell pellets from 2 L of NS3hel expression were resuspended in 50 mL of lysis buffer containing 50 mM Tris-HCl (pH 8.0), 200 mM NaCl, 5 mM β-mercaptoethanol (ΔME), 10% glycerol (buffer A), while NS3FL cell pellets were resuspended in 50 mM HEPES-NaOH (pH 7.5), 500 mM NaCl, 5 mM β-mercaptoethanol (ΔME), 5% glycerol and 0.1% *n*-octyl-β-D-glucoside (buffer B) supplemented with 1 mM PMSF. After resuspension, the lysate was treated with lysozyme (5 mg/mL) for 1 h with stirring, subjected to 15 cycles of 30 s of sonication and 30 s of resting, and treated with DNAse (20 µg/mL) for 1 h with stirring. The lysate was cleared by centrifugation at 90,000 *g* for 30 min and the supernatant was filtered through 0.22 µM filter units (Millipore, MA, USA). After filtration, the supernatant was applied at 1 mL/min to a 5 mL HisTrap HP affinity column (GE-Healthcare, Amersham, UK), previously equilibrated with 50 mL of buffer A or B. The column was washed with 10 column volumes of buffer A or B, followed by elution with an imidazole gradient (0–500 mM) performed on an ÄKTA purifier (GE-Healthcare, Amersham, UK). Gradient fractions were visualized on SDS-PAGE 12% and those containing the purified proteins were pooled and dialyzed against 2 L of buffer A or B to eliminate the imidazole. Cleavage of the NS3hel N-terminal 6xHistag was carried out overnight at room temperature mixing a volume proportion of 1∶4 (nTEV:NS3hel). The nTEV protease was previously purified and stored at a final concentration of 23 µM. Cleaved and non-cleaved proteins were separated by another affinity chromatography step, and the purified proteins were concentrated under pressure on a Stirred Ultrafiltration Cell containing a 10000 MWCO cellulose membrane (Millipore, MA, USA).

### Fluorescence spectroscopy

All fluorescence emission spectra were recorded on a Varian Cary Eclipse fluorescence spectrometer (Varian, Sydney, Australia). Slit widths of 10 nm were used for both excitation and emission and temperatures were set to 25°C. Measurements were performed using 1 µM protein concentration with excitation wavelength at 278 nm and emission spectra were recorded between 300 and 420 nm. To obtain the chemical denaturation curves, the purified proteins were incubated with increasing Gdn.HCl concentrations, from 0 to 5 M, in a buffer containing 50 mM MOPS-NaOH (pH 6.4 or 7.2), 200 mM NaCl, 5 mM βME and 5% glycerol. Fluorescence spectra at each Gdn.HCl concentration were quantified by specifying the center of spectral mass (CM):
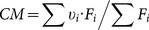
(1)where *F_i_* is the fluorescence emitted at wave number ν_i_.

The free energy of denaturation was calculated using the Gibbs equation (ΔG = -RTlnK_eq_), and the free energy change was converted empirically using the following equation:
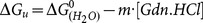
(2)where ΔG_u_ is the free energy of denaturation at each [Gdn.HCl], ΔG^o^_(H2O)_ is the free energy of denaturation in the absence of denaturant agent, and *m* is the proportionality constant that informs the solvent accessible surface area (ASA).

### Trp fluorescence quenching by acrylamide and DNA

To measure the quenching of intrinsic Trp fluorescence, aliquots of the external quencher acrylamide at 3 M were added to a 1 mL solution containing 50 mM MOPS-NaOH (pH 6.4 or 7.2), 200 mM NaCl, 5 mM βME, 5% glycerol and 1 µM of the purified proteins. Reactions were performed in triplicates and spectra were measured setting the excitation wavelength at 295 nm and emission wavelength between 325 and 355 nm. The quenching data were analyzed by the Stern-Volmer equation:
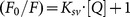
(3)which F_0_ and F are the relative unquenched and quenched intensities, respectively, K_sv_ is the Stern-Volmer constant and [Q] is the concentration of the quencher in solution.

To measure the tryptophan fluorescence quenching caused by interaction with the DNA at pH 6.4 and 7.2, aliquots of 50 µM of a ssDNA oligo(dT)_20_ were added to a 1 mL solution containing 25 mM MOPS-NaOH (pH 6.4 or 7.2), 2 mM MgCl_2_ and 1 µM of the purified proteins. Reactions were performed in triplicates and spectra were measured using the same conditions described for the acrylamide quenching.

### Circular dichroism (CD) experiments

Far UV CD measurements were carried out at increasing Gdn.HCl concentrations, from 0 to 5 M, in buffers containing 50 mM MOPS-NaOH (pH 6.4 or 7.2), 200 mM NaCl, 5 mM βME, 5% glycerol and 10 µM of NS3hel. Spectra were obtained at each Gdn.HCl concentration in a Chirascan spectropolarimeter (Applied Photophysics, London, UK) at 25°C, using a quartz cuvette with 0.01 cm path length. Final spectra were the average of three scans obtained from 190 nm to 260 nm wavelengths at a 25 nm/min speed after subtracting the spectra corresponding to the buffer base lines. Measurements of the molar ellipticity were calculated as follows:
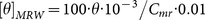
(4)where [θ]_MRW_ is the mean residue weight in degrees, C_MR_ represents the molar concentration multiplied by the number of amino acids, and 0.01 is the cuvette path length in cm.

The ellipticity values at 222 nm (θ_222_) at each Gdn.HCl concentration were used to calculate the degree of denaturation (α) according to the following equation:
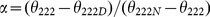
(5)where θ_222D_ and θ_222N_ are the θ_222_ values corresponding to the denatured and native species, respectively. The curves were fitted according to the linear extrapolation method proposed by Pace and Shaw [51].

### bis-ANS fluorescence

For the bis-ANS titration assays, aliquots of 40 µM bis-ANS stock were added to a 500 µL solution containing 50 mM MOPS-NaOH (pH 6.4 or 7.2), 200 mM NaCl, 5 mM βME, 5% glycerol and 1 µM of the purified proteins, obtaining final bis-ANS concentrations between 0 and 8 µM. The titrations were performed in triplicate, and bis-ANS spectra were acquired by fixing the excitation wavelength at 360 nm and the emission spectra was collected between 400 and 600 nm. To calculate the bis-ANS affinity with the protein, K_a_ values were obtained by fitting the bis-ANS binding curves using a ligand binding function available in the Sigma Plot software program v. 10.0.

To measure the changes in the bis-ANS fluorescence caused by interaction with the DNA at pH 6.4 and 7.2, aliquots of 50 µM of a ssDNA oligo(dT)_20_ were added to a 1 mL solution containing 25 mM MOPS-NaOH (pH 6.4 or 7.2), 2 mM MgCl_2_, 5 µM of bis-ANS and 1 µM of the purified proteins. Reactions were performed in triplicates and spectra were measured using the same conditions described for the bis-ANS titration assays.

### ATPase activity inhibition

The ATPase activity inhibition by the fluorescent probe bis-ANS at different pHs was determined by using a colorimetric assay based on a methodology developed by Fiske & Subbarow [52], which measures the ATP hydrolysis to ADP + Pi. In this assay, 0.1 µM of the purified proteins was incubated for 10 min at 30°C with different bis-ANS concentrations between 0.6 and 300 µM. These different bis-ANS concentrations were obtained by a 1∶2 serial dilution of stock bis-ANS in reaction buffers containing 40 mM MES-KOH (pH 6.4) or Tris-HCl (pH 7.2), 5 mM DTT, 5 mM MgCl_2_ and 100 mM KCl. The 80 µL final volume reactions were started with 1 mM ATP diluted in the same buffer, followed by 60 min incubation at 30°C. After this incubation, reactions were stopped by addition of 20 µL 50% TCA and free Pi generated was quantified after the addition of 80 µL ammonium molybdate and 40 µL reducing agent. The resulting reaction was read at 660 nm on a SpectraMax M5^e^ microplate reader (Molecular Devices, CA, USA) and relative ATPase activity at each bis-ANS concentration was obtained using the positive controls as references (reactions without bis-ANS addition). IC_50_ values were calculated by the Sigma Plot software program v. 10.0 after plotting the dose response curves between 0.6 and 300 µM.

### Fluorescence anisotropy assays

Fluorescence anisotropy assays were carried out to monitor the protein-DNA interaction at pH 6.4 and 7.2. Increasing protein concentrations (from 0 to 10 µM NS3hel and 0 to 5 µM NS3FL) were incubated with 25 nM of a ssDNA oligo(dT)_20_ labeled with a fluorescent probe (5-FAM) in 50 µL reactions containing 25 mM MOPS-NaOH (pH 6.4 or 7.2) and 2 mM MgCl_2_. After 10-min incubation, the polarization at each protein concentration was measured in a SpectraMax M5^e^ microplate reader (Molecular Devices, CA, USA) at 25°C, using 96-well black plates (Greiner Bio One, Kremsmunster, Austria). The excitation wavelength was fixed at 490 nm and emission at 520 nm, with a 515 nm cut-off. The curves were obtained in triplicate and were fitted by a ligand binding function available in the Sigma Plot software program v. 10.0.
